# Ion Channels and Electrophysiological Properties of Astrocytes: Implications for Emergent Stimulation Technologies

**DOI:** 10.3389/fncel.2021.644126

**Published:** 2021-05-20

**Authors:** Jessica McNeill, Christopher Rudyk, Michael E. Hildebrand, Natalina Salmaso

**Affiliations:** Department of Neuroscience, Carleton University, Ottawa, ON, Canada

**Keywords:** glia, physiology, ion channels, calcium, potassium, sodium, optogenetics, DREADDs

## Abstract

Astrocytes comprise a heterogeneous cell population characterized by distinct morphologies, protein expression and function. Unlike neurons, astrocytes do not generate action potentials, however, they are electrically dynamic cells with extensive electrophysiological heterogeneity and diversity. Astrocytes are hyperpolarized cells with low membrane resistance. They are heavily involved in the modulation of K^+^ and express an array of different voltage-dependent and voltage-independent channels to help with this ion regulation. In addition to these K^+^ channels, astrocytes also express several different types of Na^+^ channels; intracellular Na^+^ signaling in astrocytes has been linked to some of their functional properties. The physiological hallmark of astrocytes is their extensive intracellular Ca^2+^ signaling cascades, which vary at the regional, subregional, and cellular levels. In this review article, we highlight the physiological properties of astrocytes and the implications for their function and influence of network and synaptic activity. Furthermore, we discuss the implications of these differences in the context of optogenetic and DREADD experiments and consider whether these tools represent physiologically relevant techniques for the interrogation of astrocyte function.

## Introduction

Astrocytes comprise a heterogeneous population of macroglial cells that are the most abundant neural cell type in the central nervous system (CNS). Similar to both neurons and oligodendrocytes, astrocytes arise from the neural stem cell pool (Sloan and Barres, 2014). The process of gliogenesis in rodents begins around embryonic day 16–18 with the majority of cortical astrogliogenesis likely occurring in the postnatal period where a substantial increase in glial numbers are observed during the second and third postnatal weeks (Abney et al., 1981; Qian et al., 2000; Bushong et al., 2004; Freeman, 2010). The extensive morphological and functional heterogeneity of astrocytes is in part driven by their place of birth and neuronal neighbors during the course of development (Lanjakornsiripan et al., 2018; Bayraktar et al., 2020). This may be mediated, in part, through the release of specific neurotransmitters or neurotrophic factors from these nearby neurons. At least in cortical development, neuronal heterogeneity induces differential astroglial phenotypes (Bayraktar et al., 2020).

Morphologically, astrocytes can take many forms, though they are perhaps best known for their protoplasmic shape; a smaller soma surrounded by numerous processes that extend outwards (Sofroniew and Vinters, 2010), giving them a “star-like” shape for which they are named. In addition to these protoplasmic astrocytes, which predominantly exist in gray matter, there are fibrous astrocytes which are found throughout the white matter (Sofroniew and Vinters, 2010). The somata of these cells orient themselves in perpendicular rows between the axon bundles while their processes make connections to nodes of Ranvier (Oberheim et al., 2009). These two dominant morphological types of astrocytes are by no means an exhaustive list; velate astrocytes of the olfactory bulb and cerebellum, Bergmann glia of the cerebellum, Müller glia in the retina, pituicytes of the neurohypophysis, radial glia, and Gomori astrocytes of the hypothalamus all represent morphologically-distinct classes of astrocytes (Verkhratsky and Nedergaard, 2018; Khakh and Deneen, 2019).

Astrocytes display even greater diversity in their functional roles. Previously believed to only provide structural support to neurons, it is now well-established that astrocytes are key regulators of CNS homeostasis. Some of these homeostatic functions include the buffering of ions like potassium (K^+^), sodium (Na^+^), and protons (H^+^), and the regulation of neurotransmitters. Astrocytes form an integral part of the tripartite synapse; their processes encompass the synapse, allowing them to remove excess neurotransmitters from the cleft. This is particularly important for the excitatory neurotransmitter, glutamate: astrocytes are responsible for removing and breaking down almost all central extracellular glutamate (Mahmoud et al., 2019). These glutamatergic transporters are also critical for modulating neuronal plasticity; for example, downregulation of the glutamate-1 transporter has been shown to impair long-term potentiation (LTP; Li Y.-K. et al., 2012).

In addition, astrocytes contribute to CNS homeostasis by forming an integral part of the blood-brain barrier; their endfeet surround the cerebral capillaries as part of the neurovascular unit (Liedtke et al., 1996; Wilhelm et al., 2016). Thus, they play a key role in modulating the entry of molecules into the CNS; permitting access for essential substances like nutrients while preventing access of potentially harmful agents like oxidants (Wilhelm et al., 2016). Astrocytes are also crucial for regulating pH levels, modulating oxidative stress, and providing energy substrates to neurons. For example, astrocytes are key metabolizers of glucose, the main source of energy (ATP) production in the brain (Prebil et al., 2011).

Astrocytes are also key players in CNS injury response, undergoing morphological and functional changes in a process known as reactive astrogliosis. Their response differs according to the extent and cause of the injury and includes molecular, morphological, and physiological changes (Sofroniew and Vinters, 2010). Reactive astrocytes can produce neurotoxic or neuroprotective effects. Two distinct classifications of reactive astrocytes, termed “A1” and “A2” (neurotoxic and neuroprotective, respectively) have recently been characterized (Liddelow et al., 2017), though it is likely more phenotypes exist.

Despite the accumulating evidence demonstrating the extensive regional and sub-regional diversity of astrocytes, there remains very little understanding of how the electrophysiological properties of astrocytes may diverge across these subpopulations. A large body of evidence in the field suggests that astrocytes may not be as “electrically silent” as previously believed, so characterizing differences in these electrophysiological properties will be important for understanding the functional differences of astroglial cells. Moreover, the increased use of technologies such as designer receptors activated by designer drugs (DREADDs) and optogenetics in astrocytes, augments the need to understand the physiological properties of astrocytes as these properties will be critical for the future application of these tools to this cell population. A greater knowledge of astrocyte physiology will inform experimental design, determine the physiological relevance (or not) of specific electrical stimulation experiment(s) and help with acknowledging the limitations of each. In the remainder of this review, we will highlight the regional differences in astrocyte physiology, and discuss the implications for optogenetic and DREADD manipulation of astrocytes.

## Heterogeneity of Astrocyte Membrane Potential, Resistance and Current Patterns Under Basal and Reactive Conditions

### Astrocytes Are Electrically Active Cells

Though neurons are the main excitable cell type of the brain, astrocytes are not “electrically silent” cells. Astrocytes have a hyperpolarized membrane (Du et al., 2016) that typically rests below that of neurons (see section below; Bolton et al., 2006; Zhou et al., 2006), in contrast to the majority of non-excitable cells that have relatively depolarized membrane potentials. Though they cannot generate an action potential, astrocytes are able to respond biochemically to stimuli within their environment, especially ions and neurotransmitters. Astrocytic membranes are rich with several cation and anion channels (including both voltage-dependent and voltage-independent channels), which help with the regulation of ions such as Na^+^, K^+^, Ca^2+^ and Cl^−^, in addition to contributing to the resting membrane potential (RMP), resting conductance and intracellular signaling within astrocytes (Parpura et al., 2011; Ryoo and Park, 2016). Several of these channels have permeability properties that are independent of voltage (i.e., TWIK 1, TREK 1—see “Voltage-Independent K^+^ Channels Contribute to Passive Conductance” section), but many are voltage-dependent (such as delayed–rectifying potassium channels—see “Voltage-Dependent K^+^ Channels Have Distinct Subcellular Localization” section). Therefore, astrocytes are a dynamic cell type with functional and signaling properties that vary with changes in membrane potential. In addition to ion-permeable channels, astrocytes also express several electrogenic transporters to help facilitate the exchange of ions across their membrane. For example, the Na^+^-K^+^-ATPase pump exchanges three Na^+^ ions out for every two K^+^ ions in, and the Na^+^-K^+^-2Cl^−^-cotransporter pump exchanges Na^+^, K^+^, and Cl^−^, with an accompanying influx of water into the cell (Bellot-Saez et al., 2017). These ion channels and transporters represent an important facet of astrocyte physiology; astrocytic Ca^2+^ signaling represents another.

Calcium signaling in astrocytes plays an important role in facilitating the bidirectional communication between neurons and astrocytes at the synapse (for a more complete review on neuron-astrocyte interactions at the synapse, see Allen and Eroglu, 2017). Astrocytes express a plethora of ionotropic and metabotropic receptors, enabling a diverse set of responses to neurotransmitters such as glutamate, serotonin, dopamine, and GABA (Verkhratsky et al., 2019). Neurotransmitter binding to astrocytes can induce intracellular Ca^2+^ signals, and the magnitude, localization and time course of these signals vary significantly depending on the stimulus and synaptic network involved (Araque et al., 2014). However, this neuron to astrocyte communication is not the only form of interaction between these cell types. Astrocytes are able to modulate neuronal activity through the release of several active factors such as glutamate, ATP and D-serine in a process known as gliotransmission (Parpura et al., 1994; Cotrina et al., 2000; Henneberger et al., 2010; Araque et al., 2014; Perez et al., 2017). This process is partly mediated by intracellular Ca^2+^ signaling pathways (Araque et al., 2014). The release of these gliotransmitters from astrocytes is known to regulate synaptic transmission and plasticity. Changes in the frequency of miniature and spontaneous excitatory postsynaptic currents (EPSCs) and inhibitory postsynaptic currents (IPSCs), and the modulation of both LTP and long-term depression (LTD) have all been observed following the release of gliotransmitters from astrocytes across several different brain regions including the hippocampus, cortex and cerebellum (Kang et al., 1998; Brockhaus and Deitmer, 2002; Takata et al., 2011; Navarrete et al., 2012; Araque et al., 2014). Calcium signaling in astrocytes has also been shown to stimulate the Na^+^-K^+^-ATPase pump, leading to a decrease in extracellular K^+^ and subsequent neuronal hyperpolarization and suppression of baseline excitatory activity (Wang et al., 2012).

### Astrocytes Display a Highly Negative Resting Membrane Potential

Compared to their neuronal counterparts, astrocytes display a more hyperpolarized, or negative, RMP (Bolton et al., 2006). While neuronal membranes typically rest at between approximately −50 mV and −70 mV (Zaitzev et al., 2012; Shen et al., 2018; Fernandez et al., 2019), the RMP of astrocytes is typically lower. However, specific astrocyte RMP values vary substantially across the CNS. For example, mature astrocytes of the CA1 region of the hippocampus have an RMP of about −80 mV (Tang et al., 2009; Deemyad et al., 2018), whereas astrocytes of the optic nerve have an average RMP of −62 mV (Butt and Jennings, 1994). A comparison between telencephalic astrocytes found that those of the *stratum oriens* and *stratum pyramidale* regions of the hippocampus had a significantly more negative (RMP of −90 mV) average membrane potential compared to those of the layers V and VI of the cortex (with an RMP of about −85 mV; Mishima and Hirase, 2010).

There is also extensive heterogeneity of astrocytes within the *same* region; astrocytes of the optic nerve have an RMP ranging from −25 to −80 mV (Bolton et al., 2006) whereas those in the ventral tegmental area (VTA) ranged from −60 to −90 mV (Xin et al., 2019). The hippocampus has proven to have a diverse pool of astrocytes; astrocytes in this area have RMPs ranging from −80 mV in the CA1 and *stratum radiatum* subregions (Zhong et al., 2016; Deemyad et al., 2018) to a more negative RMP (−90 mV) in the *stratum oriens* and *stratum pyramidale* (Mishima and Hirase, 2010).

In neurons, the RMP is important for setting the threshold, propensity, and frequency of action potentials. In astrocytes, which lack action potentials, the highly negative (hyperpolarized) RMP is critical for enabling and regulating homeostatic functions such as K^+^ buffering and even neurotransmitter reuptake (Zhou et al., 2006; Ryoo and Park, 2016). Variability in astrocytic RMPs throughout the CNS may therefore reflect differences in (some) astrocytic functions.

**Figure 1:**
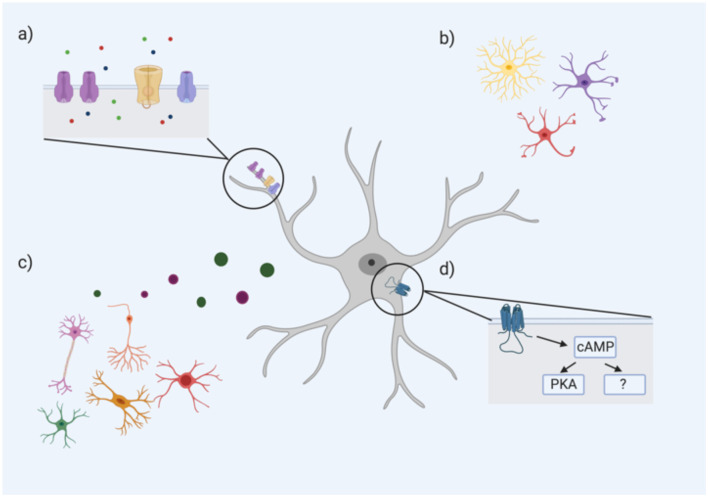
An astrocyte’s resting membrane potential (RMP) is likely influenced by multiple extrinsic and intrinsic factors including **(A)** ion channel subtype expression and density, particularly Ca^2+^, Na^+^, and K^+^ channels (ions represented by green, red, and blue circles, respectively); **(B)** astrocyte morphology; **(C)** neighboring cells including neurons, oligodendrocytes, microglia, and other astrocytes through the release of neurotransmitters, gliotransmitters, and other factors; **(D)** intracellular astrocyte signaling cascades such as the cAMP pathway. cAMP PKA-dependent and PKA-independent mechanisms have been proposed to influence astrocyte RMP (Bolton et al., 2006).

The underlying reason(s) for differences in astrocyte RMP across brain regions and subtypes have not been fully elucidated, but it is likely a result of a complex interplay between numerous extrinsic and intrinsic factors. An astrocyte’s immediate environment and neuronal input may drive heterogeneity in RMP values across the CNS. The morphological characteristics of individual astrocytes may also play a role in determining RMP. For example, a heavily branched astrocyte with numerous processes and greater membrane surface area may have a higher number of ion channels, particularly leak channels (i.e., K^+^), that could in part explain some of the differences in astrocyte RMP (relationships between morphology, RMP heterogeneity, and differential astrocytic Ca^2+^ signaling- *are further discussed in* *“Ca^2+^ signaling pathways in astrocytes”*). However, as specific morphological classes of astrocytes have not been linked with particular ranges of RMPs, it is highly probable that several other factors also influence the RMP of an astrocyte, such as intracellular signaling pathways (see Figure 1).

One intracellular signaling cascade that may influence the RMP of astrocytes is the cAMP/PKA pathway. Bolton et al. (2006) demonstrated that incubation of astrocytes with a cAMP analog (to activate adenylate cyclase) hyperpolarized their mean RMP. The addition of a PKA inhibitor caused a significant depolarization of the astrocytic membrane, but this effect was only partially reversed with the cAMP analog, suggesting that cAMP influences astrocytic RMP *via* both PKA-dependent and—independent pathways (Bolton et al., 2006). Therefore, the variability of astrocytic RMP may be mediated by differences in intracellular cAMP/PKA signaling. In astrocytes, this cAMP/PKA pathway has been linked to changes in cell morphology and gene expression. A recent study found that over 6,000 astroglial gene transcripts were differentially regulated by cAMP signaling; gene ontology revealed associations with pathways controlling antioxidant activity, cell metabolism, and ion transporters (Paco et al., 2016). For example, numerous ion channels, pumps and transporters such as Kcn2 (for K^+^) and ATP2a2 (for Ca^2+^) were all upregulated by cAMP (Paco et al., 2016). Given the critical role of cAMP in these various functions, the differences in astrocyte RMP may be an electrophysiological hallmark of critical differences in cAMP signaling of astrocytes between and within regions (see Figure 1).

### Astrocytes Are Characterized by Low Membrane Resistance

In addition to their negative RMP state, astrocytes typically have a dramatically lower membrane input resistance than neurons under basal conditions (Zhou et al., 2006; Ma et al., 2014; Du et al., 2015; Xin et al., 2019), suggesting a relatively high overall permeability to ions at rest. The low input resistance of astrocytes makes it particularly challenging to study their biophysical properties using electrophysiological approaches (for example, the low membrane resistance can cause a large portion of the voltage drop to occur across the electrode tip rather than across the cell membrane change; Ma et al., 2014). Nonetheless, the available data suggests astrocytic membrane resistance differs across brain regions, and even within the same region. For example, VTA astrocytes have, on average, a significantly lower membrane resistance compared to those of the cortex or hippocampus (approximately 1 MΩ in the VTA compared to approximately 3 MΩ in the cortex and hippocampus; Xin et al., 2019), though no differences have been noted between cortical and hippocampal astrocytes (Mishima and Hirase, 2010; Xin et al., 2019). However, several studies have found differences in membrane resistance amongst astrocytes within the hippocampus (Isokawa and McKhann, 2005; Zhong et al., 2016), suggesting that the membrane resistance of only some hippocampal astrocytes are comparable to those in the cortex. In one study, two distinct electrophysiological phenotypes of astrocytes were identified in the *stratum radiatum*; one subclass was defined by a variable input resistance with an overall mean input resistance significantly higher than the other subclass (Zhong et al., 2016).

Precisely what these differences mean is not fully understood, however, because input resistance is inversely proportional to overall ion permeability across the membrane, these differences might represent variability in the capacity of astrocytes to conduct/transport ions in and out of the cell. Therefore, differences in membrane resistance could offer significant insight into the ability of astrocytes to buffer various ions as well as resultant downstream intracellular signaling pathways driven by these ions.

It is also important to consider how morphology, and specifically, membrane surface area, may influence membrane resistance. An increase in the number or length of astrocytic processes and therefore, membrane surface area, will lead to an increase in leak channels (if channel density is equal across these membrane processes), and thus an increase in overall membrane conductance. An increase in membrane conductance will directly result in a decrease in membrane resistance. While differences in morphology alone may be insufficient to explain the heterogeneity in astrocytic membrane resistance, it is likely that it is a contributing factor.

Challenges to the CNS have also been noted to influence membrane resistance in astrocytes, though these changes are variable and appear to depend on the type and extent of the challenge. Following a unilateral entorhinal cortex lesion, astrocytes of the denervated layer in the dentate gyrus had an increase in membrane resistance that persisted for up to 10 days post-lesion (Schröder et al., 1999). Another study observed changes of membrane resistance in some astrocytes, but not others, following incubation with high [K^+^], a model of early astrocyte activation (Neprasova et al., 2007). A decrease in the membrane resistance of hippocampal astrocytes in slice was noted following exposure to ammonium (a model of hepatic encephalopathy; Stephan et al., 2012).

In a cortical freeze lesion model, changes in the membrane resistance of astrocytes were noted though these changes varied significantly depending on the relative location of the astrocytes to the injury site (Bordey et al., 2001). Increased membrane resistance was noted in layer I astrocytes of a lesioned cortex compared to controls but astrocytes in the “hyperexcitable” zone (characterized by the epileptiform activity of neurons upon stimulation) showed virtually no changes in membrane resistance (Bordey et al., 2001). Additionally, this model induced a proliferative zone; an area surrounding the lesion characterized by proliferating astrocytes (Bordey et al., 2001). Interestingly, astrocytes in this proliferative zone had a mean membrane resistance that was significantly higher than astrocytes of the hyperexcitable zone under both control and lesioned conditions (Bordey et al., 2001).

The changes in astrocytic membrane resistance following disturbances to the CNS may represent compensatory mechanisms. A low membrane resistance, such as the one typically seen in astrocytes, suggests increased ionic permeability across the astrocyte’s membranes. The initial increase in input resistance following a unilateral entorhinal cortex lesion (Schröder et al., 1999) suggests a reduced ability of ion conduction across the membrane in the proliferative zone. This might represent a mechanism to help regulate ion homeostasis during times of CNS perturbations. On the contrary, this increase in membrane resistance could also represent a mechanism that perpetuates CNS damage. If an increase in membrane resistance corresponds to a reduced ability to transport ions across the membrane, this could mean a reduced ability to maintain extracellular ion homeostasis, thus causing further damage.

These injury-induced changes in astrocyte membrane resistance may correspond with the changes in astrocyte morphology that characterizes reactive astrogliosis. Following CNS injury, hypertrophy of astrocytes occurs in levels correlative to the severity of the injury (Sofroniew and Vinters, 2010). Retraction of astrocytic processes and a hypertrophied cell soma means a smaller membrane surface area (and potentially fewer leak channels), which could explain an increase in membrane resistance. As astrocytes closer to the site of injury tend to undergo greater hypertrophy, this could partially explain why the membrane resistance of astrocytes might vary across different zones of the injury site. In the case of localized CNS damage (such as a lesion or ischemic event), the location of astrocytes relative to the damage may represent a significant factor in determining whether the membrane resistance increases, decreases, or remains the same. In Bordey et al.’s (2001) study, the physiological changes differed significantly amongst the different zones of the injury, suggesting this may be an important factor influencing astrocyte physiology under reactive conditions.

Reactive astrogliosis has long been recognized as a process that induces morphological, molecular and physiological changes. Data from studies that have induced CNS damage (Schröder et al., 1999; Bordey et al., 2001; Neprasova et al., 2007; Stephan et al., 2012) demonstrate clearly that the basic electrophysiological properties of astrocytes (i.e., membrane resistance) also change in response to perturbations in the CNS, though in many contexts, these changes are not well characterized and understood. As the perspective of astrocyte heterogeneity continues to develop, under basal and reactive conditions, it will be essential for the perspective of astrocyte electrophysiology to do the same.

### Electrophysiological Properties of Astrocytes Across Development

Further contributing to the complexity of astrocyte physiology is that many of the other basic electrophysiological properties of astrocytes such as their membrane potential change across development (Zhou et al., 2006; Zhong et al., 2016). One study found that neonatal astrocytes (P1–P3) of the *stratum radiatum* had a more negative membrane potential compared to mature astrocytes (*P* > 21) of the same region (approximately −85 mV and −80.9 mV, respectively; Zhong et al., 2016). These changes across development highlight the caution that must be taken when generalizing electrophysiological data across studies and across timepoints.

It is currently unknown what drives these changes in the electrophysiological properties of astrocytes throughout development. In the cortex, morphological and functional heterogeneity of astrocytes is influenced greatly by neuronal heterogeneity *via* the release of specific neurotransmitters, ions, and neurotrophic factors (Verkhratsky and Nedergaard, 2018; Bayraktar et al., 2020); it is likely that this is also the case for the electrophysiological features of astrocytes. It is also possible these changes reflect shifts in the expression of ion transporters. For example, the K^+^-Cl^−^- cotransporter (KCC2) helps modulate Cl^−^ levels in neurons through the export of 1 K^+^ and 1 Cl^−^ across the membrane (Annunziato et al., 2013). An increase in the neuronal expression of KCC2 early in development is believed to drive the shift of GABA from an excitatory to an inhibitory neurotransmitter (Moore et al., 2019). However, KCC2 is also expressed in astrocytes (Annunziato et al., 2013; Rurak et al., 2020), and this expression increases across development (Rurak et al., 2020). Perhaps the increase in KCC2 expression in astrocytes, and subsequent changes in intracellular K^+^ and Cl^+^ levels and reversal potentials, is what drives the changes in the electrophysiological properties such as RMP over development. Moreover, increased ion transport across the membrane *via* transporters such as KCC2 could also influence the input resistance of astrocytes. Developmental changes in transporter expression may represent one potential mechanism that drives changes in astrocyte electrophysiology across the lifespan.

It is clear from the literature that astrocytes differ tremendously in many of their basic electrophysiological properties such as their RMP, membrane resistance, membrane currents, and selective ion permeability. The literature also shows that these properties differ significantly at both the regional and sub-regional levels. There remains a large gap in knowledge about the relationship between the specific electrophysiology properties of astrocyte subpopulations and how these properties correspond to their morphological, biochemical, and functional properties. It is likely that future studies on astrocyte electrophysiology, will result in new methods for classifying and modulating signaling within astrocyte subtypes.

### Astrocyte Electrophysiology: Convergence Across Species

The evidence presented thus far for the heterogeneity of astrocyte electrophysiology is primarily derived from studies utilizing *in vitro* or *in vivo* rodent models. However, whether the electrophysiological properties of astrocytes are conserved across species is not well characterized. Furthermore, while rodent astrocytes appear to exhibit extensive heterogeneity in their electrophysiology, it is unknown whether (or to what extent) human astrocytes show similar heterogeneity in *their* electrophysiological properties. However, it is well established that rodent and human astrocytes do differ in many of their characteristics (Oberheim et al., 2009; Dossi et al., 2018; Miller, 2018).

Morphological and transcriptional analyses have revealed differences between astrocytes across species. For example, several studies have demonstrated that human astrocytes tend to exhibit larger soma with a greater number of processes compared to their rodent counterparts (Oberheim et al., 2006, 2009; Zhang et al., 2016). Transcriptional differences have also been noted; one study found over 600 genes enriched in human astrocytes that were not enriched in mouse astrocytes (Zhang et al., 2016). Given this divergence between species, it is possible that human astrocytes also differ in terms of their electrophysiological properties.

Few studies have been conducted that evaluate the electrophysiological properties of human astrocytes. Early studies of human astrocytes measured comparable RMPs to those observed in rodents (Bordey and Sontheimer, 1998; O’Connor et al., 1998; Hinterkeuser et al., 2000). One of these studies noted a higher membrane capacitance in human astrocytes (Bordey and Sontheimer, 1998), which is perhaps expected given the larger surface area of human astrocytes (Bedner et al., 2020). Interestingly though, this study did note a high input resistance (Bordey and Sontheimer, 1998). However, there is contention over whether these early studies successfully analyzed astrocytes, or whether they had actually identified NG2+ glial cells (Bedner et al., 2020). A more recent study of hippocampal astrocytes from patients with temporal lobe epilepsy revealed they exhibited a passive conductance and an RMP, membrane resistance and capacitance similar to rodent astrocytes of the same region (Bedner et al., 2015, 2020). This suggests that some electrophysiological properties of astrocytes are, in fact, conserved between rodents and humans (Bedner et al., 2020). However, there lacks sufficient studies/evidence to draw any strong conclusions.

Given the heterogeneity of astrocytes in the rodent brain, it is possible that a similar diversity in electrophysiological properties exists in human astrocytes. Studies involving direct comparisons across species will also be critical; this is particularly important because of the observed differences in species morphology, and the link between morphology and membrane capacitance. Any differences between rodent and human astrocyte electrophysiology could potentially be explained by differences in morphology. However, one study did show comparable membrane capacitance values between mouse and human astrocytes (Bedner et al., 2015, 2020), which could mean other factors are influencing human astrocyte physiology such as the density of ion channel expression. At this time, too few studies have been conducted to reach reliable conclusions as to if, and to what extent, human astrocyte electrophysiology differs from their rodent counterparts.

An additional caveat of astrocyte electrophysiology research is the extensive differences in the experimental protocol. Although *in vitro* (i.e., cell culture and slice) and *in vivo* data suggests comparable findings across experimental paradigms, few, if any, studies have directly compared electrophysiological properties of astrocytes from culture, slice and *in vivo* samples. Therefore, this calls for caution when interpreting and extrapolating electrophysiological data across paradigms.

## Astrocytes are Key Regulators of K^+^ Homeostasis

### Astroglial K^+^ Spatial Buffering Mediated Through K_ir_4.1 Subtype

The combination of a highly negative RMP and a low membrane resistance make astrocytes particularly well suited for buffering potassium (K^+^; Du et al., 2015), one of their most critical homeostatic functions within the CNS. The extracellular concentration of K^+^ ([K^+^]_o_) rests at approximately 3.0 mM, and is critical in establishing the RMP of both neurons and astrocytes (Anderson et al., 1995; Bellot-Saez et al., 2017). Interestingly, the average RMP of astrocytes is close to the equilibrium potential of K^+^ (Somjen, 1979; Guatteo et al., 1996), thus reflecting a high resting conductance for the ion (Somjen, 1979; Dallérac et al., 2013).

Changes in [K^+^]_o_ can be indicative of increased neuronal activity (Neprasova et al., 2007). Increases in [K^+^]_o_ occur following neuronal excitation whereby K^+^ clearance from the neuron is used to co-transport Na^+^ ions out of the cell following periods of high action potential-mediated Na^+^ influx (Hertz and Chen, 2016). Thus, regulation and uptake of extracellular K^+^ is essential in maintaining a homeostatic balance within the CNS. While both neurons and astrocytes are capable of regulating K^+^ levels, this function is typically associated with astroglial cells. Several mechanisms of K^+^ maintenance have been identified including passive spatial buffering and uptake mediated through active transporters.

The concept of K^+^ spatial buffering was first proposed decades ago (Walz, 1982; Verkhratsky and Nedergaard, 2018). In the proposed model, K^+^ enters astrocytes *via* K^+^-permeable membrane channels and diffuses to areas of lower K^+^ concentration in the glial network *via* gap junctions connecting the glial syncytium (Higashi et al., 2001; Verkhratsky and Nedergaard, 2018). This occurs without additional energy requirements (Orkand et al., 1966; Bellot-Saez et al., 2017). The initial uptake of extracellular K^+^ prior to its redistribution throughout the glial syncytium is mediated through many subtypes of K^+^-permeable channels, including both voltage-dependent (i.e., inward-rectifying and Kv families) and independent (i.e., two-pore domain or “leak” family, see next section) K^+^ channels. Each of these families of channels express several subtypes including TREK 1, TWIK 1, and Kv 3.4 and 4.3 (this is not an exhaustive list but will be the focus in the remainder of this section). Of the variants of voltage-dependent K^+^ channels, the inward-rectifying K^+^ channels are a family consisting of 16 channels, subdivided into seven subfamilies (Bellot-Saez et al., 2017). The K_ir_4.1 subtype, a weakly inward-rectifying K^+^ channel, is the predominant subtype expressed on astrocytes (Kofuji and Newman, 2004; Brasko et al., 2017).

The robust expression of the K_ir_4.1 subtype is believed to contribute to the high resting conductance of K^+^ in astrocytes (Tang et al., 2009). However, expression of the K_ir_4.1 subtype within astrocytes is variable; the channel is expressed in the spinal cord (Olsen et al., 2006), deep cerebellar nuclei, Müller glia of the retina as well as a subset of the hippocampus, but not in all astrocytes found within the hippocampus and white matter (Poopalasundaram et al., 2000; Higashi et al., 2001; Rurak et al., 2020). In a comprehensive analysis of K_ir_4.1 subtype expression in glial cells, K_ir_4.1 immunoreactivity was enriched in astrocytic processes wrapped around blood vessels (Hibino et al., 2004) and at synapses. Enrichment of K_ir_4.1 at blood vessels was also noted in human tissue (Tan et al., 2008).

The channel subtype was observed on astrocytes in several regions, including the forebrain, midbrain, and hindbrain, albeit to varying degrees (Higashi et al., 2001). The percentage of total synapses covered by K_ir_4.1-positive processes varied substantially between brain regions, with over 60% of synapses covered in regions such as the entorhinal cortex, the superior and inferior colliculi and the pontine nucleus, but only 30%–60% of synapses covered in regions such as the anterior dorsal nucleus and lateral nuclei of the thalamus and the interpeduncular nucleus. Other regions, like the mitral cell layer of the olfactory bulb, exhibited very little K_ir_4.1-positive processes surrounding synapses (Higashi et al., 2001).

The expression of the K_ir_4.1 subtype is also variable within the cortex. Benesova et al. (2012) identified distinct subpopulations of astrocytes within the cortex that differed in their extent of swelling following oxygen-glucose deprivation. These subpopulations were (nearly) uniformly distributed across each layer of the cortex but varied significantly in gene expression of several K^+^-related channels, including K_ir_4.1. There was approximately a 1.5 log difference in gene expression between the subpopulations (Benesova et al., 2012). In contrast, another study found differences in K_ir_4.1 immunoreactivity between layers of the cortex; there was greater K_ir_4.1 subtype expression in cortical layers II and III compared to layers IV–VI, though this study did not distinguish between (possible) subtypes of astrocytes (Higashi et al., 2001).

The subregional diversity of astrocytic K_ir_4.1 subtype expression has been noted in other regions including the olfactory bulb and the hippocampus (Higashi et al., 2001). Each layer of the olfactory bulb, for example, has varying expression of the K_ir_4.1 subtype subunit, with high expression in layers such as the glomerular layer, and much lower expression in layers such as the olfactory nerve and mitral cell layers (Higashi et al., 2001). A similar pattern was seen in the hippocampal layers; almost no K_ir_4.1 was seen in the dentate gyrus but there was astroglial expression of the channel in the CA layers (Higashi et al., 2001). Moreover, co-localization of the K_ir_4.1 subtype with glial fibrillary acidic protein (GFAP), an intermediate filament protein commonly used as a marker for astrocytes, showed differences between each layer of the olfactory bulb (Higashi et al., 2001), further demonstrating the molecular and physiological heterogeneity of astrocytes.

There is some evidence to indicate heterogeneous subcellular localization of K_ir_4.1 in astrocytes. One study of cerebellar glia found K_ir_4.1 expression in the radial processes of Bergmann glia in the Purkinje cell layer, whereas astrocytes of the granule cell layer expressed K_ir_4.1 in both the processes and somata (Brasko et al., 2017). In contrast, Muller glia of the retina preferentially express K_ir_4.1 in their perivascular endfeet vessels (Kofuji et al., 2002). Similarly, K_ir_4.1 is expressed in endfeet and fine processes of astrocytes within the rat optic nerve (Kalsi et al., 2004). The function of K_ir_4.1 may be mediated by its subcellular localization; at perivascular endfeet, the ion channel may be important for regulating K^+^ in the blood vessels, but those localized at the processes or somata may be more important for regulating K^+^ levels of the astrocyte itself. Nonetheless, the heterogeneity of subcellular K_ir_4.1 expression across different regions further emphasizes the extensive diversity of astrocyte electrophysiology.

The variability of astrocytic K_ir_4.1 subtype expression further demonstrates the physiological heterogeneity of astrocytes. Whilst differing levels of the K_ir_4.1 subtype are not necessarily indicative of differing capabilities of K^+^ regulation, it does suggest, at the very least, that astrocytes of varying brain regions utilize alternative mechanisms to regulate K^+^. It appears astrocytes of the *same* region may also exhibit alternative mechanisms for K^+^ regulation as they also display differing levels of the K_ir_4.1 subtype; and since K_ir_4.1 appears to be particularly important in generating the RMP of astrocytes, it is possible that astrocyte subtypes with different membrane potentials express different levels of this K^+^ channel. That astrocytes might differ concomitantly in distinct features (i.e., morphological, physiological, functional) demonstrates the complexity of astrocyte heterogeneity, and the importance of further understanding the physiological diversity of this unique cell population.

### Voltage-Independent K^+^ Channels Contribute to Passive Conductance

The two-pore domain, voltage-independent K^+^ channels (K_2P_) are thought to contribute substantially to the electrophysiological properties of astrocytes (Ryoo and Park, 2016; Verkhratsky and Nedergaard, 2018). This group of “leak channels” is a 15-member family of which at least three channel subtypes have been identified in astrocytes (Seifert et al., 2009; Du et al., 2016). TREK 1, TREK 2, and TWIK 1 have been observed in astrocytes of the hippocampus (Seifert et al., 2009; Du et al., 2016), cortex (Gnatenco et al., 2002), and forebrain (Cahoy et al., 2008), though it is likely that these channels are expressed in astrocytes throughout the CNS.

Despite their voltage-independence, K_2P_ channels mediate currents at a wide range of membrane potentials and are believed to contribute to the RMP of neurons and astrocytes (Ryoo and Park, 2016; Verkhratsky and Nedergaard, 2018). In the hippocampus, passive conductance (that is a linear current-voltage relationship) in astrocytes was reduced following a pharmacological blockade and shRNA-mediated knockdown of TREK 1 and TWIK 1 (Zhou et al., 2009; Mi Hwang et al., 2014), suggesting a contributory role of these channels to the passive conductance and K^+^ uptake observed in astrocytes (Seifert et al., 2009). However, there is some contradictory evidence to this as the genetic deletion of TWIK and/or TREK 1 did not alter passive conductance in hippocampal astrocytes (Du et al., 2016). If TWIK 1 and TREK 1 are involved in passive conductance, then differences in astrocytic conductance throughout the CNS suggests potential variability in the expression of these leak channels throughout the brain. Since the passive conductance of astrocytes is thought to be the reason for their ability to buffer K^+^ (Tang et al., 2009), this suggests TREK 1 and TWIK 1 might also play a contributory role in the ability of astrocytes to buffer K^+^. Further research is needed to determine if indeed the expression of TREK 1 and TWIK 1 influence K^+^ buffering, and to what extent. It is also possible (and likely) these channels are important in determining other functions in astrocytes, further highlighting the need for more research.

### Voltage-Dependent K^+^ Channels Have Distinct Subcellular Localization

Beyond K_ir_4.1, several other types of voltage-dependent K^+^ (K_V_) channels, with heterogeneous biophysical properties, have been identified in astrocytes (Verkhratsky and Nedergaard, 2018). As a first example, delayed rectifying K^+^ currents have been observed in astrocytes from the spinal cord, hippocampus, cerebellum, and cortex (Bordey and Sontheimer, 2000). The delayed rectifying K^+^ current ion channel subtype (K_D_) that mediates these currents has outward rectification and a higher conductance capability at potentials more positive than −50 mV. Transient “A”-type currents are a second type of K^+^ current present in astrocytes of the cerebrum, hippocampus, spinal cord, and the optic nerve (Sontheimer, 1994). The A-type channels are rapidly activating and inactivating and also require hyperpolarization to remove the tonic inactivation before they can activate (Sontheimer, 1994; Verkhratsky and Nedergaard, 2018). There are also additional subtypes of inward-rectifying K^+^ channels beyond K_ir_4.1 that have been characterized in astrocytes (Bekar et al., 2005). In astrocytes, these voltage-gated K^+^ channels are thought to play a role in modulating membrane potential; blockade of K_v_ channels in cortical astrocytes diminished their ability to repolarize (Wu et al., 2015). Blockade of these channels also reduced the influx of Ca^2+^, suggesting a role of these channels in the regulation of Ca^2+^ entry into astrocytes (Wu et al., 2015).

Various voltage-dependent K^+^ channels are expressed in astrocytes; Kv1.1 and Kv1.6 are seen in cortical mouse astrocytes (Smart et al., 1997); Kv1.5 has been observed in the spinal cord and brains of rats, as well as in gliomas of human patients (Preussat et al., 2003). Kv1.3 is also expressed in human gliomas (Preussat et al., 2003). In addition, the subtype and subunit composition of voltage-gated K^+^ channels differ across astrocyte subpopulations, at least in the hippocampus. One study found three families of K^+^ channels, Kv4, Kv3, and Kv1 that each contributed a different percentage of the A-type currents observed in the hippocampus (about 70, 10 and 5%, respectively; Bekar et al., 2005). These K^+^ channels also exhibit distinct subcellular localization in astrocytes; the Kv3.4 subtype was expressed primarily in the processes whereas the Kv4.3 was found localized to the somata (Bekar et al., 2005). The functional implications of this have not been fully elucidated, but do suggest that there may be distinct responses of the subcellular components in astrocytes to changes in voltage. Whether the differential expression of Kv families in hippocampal astrocytes is comparable to astrocytes of other brain regions has not yet been characterized. However, the variability in astrocytic membrane potential, which is partially modulated through voltage-gated K^+^ channels, suggests there is likely variability in the expression of these channels across astrocytes of other areas.

Like Kv3.4 and Kv4.3, the Kv1.3 and Kv1.6 subtypes exhibit distinct subcellular localization. In rat astrocytes, Kv1.3 is expressed on the Golgi apparatus and the Kv1.6 subtype is on the endoplasmic reticulum (Zhu et al., 2014), thus reiterating the heterogeneous nature of astrocytes and astrocyte physiology.

At least one study has demonstrated that the cAMP/PKA pathway might influence the RMP of astrocytes (Bolton et al., 2006). It is possible that the cAMP pathway regulates RMP through the modulation of voltage-gated K^+^ channels. In fact, one study did find that treating cultured astrocytes with activators of the cAMP pathway was sufficient to modulate the expression (both up-and downregulation) of several potassium channels (Paco et al., 2016). Activation of this pathway through neuronal input, intracellular (astrocyte) signals, environmental factors, or some combination of these, might influence the temporal and spatial expression of these channels, and ultimately the RMP of an astrocyte. Particularly over the course of development, these neuronal inputs, signals and, environmental influences vary greatly from region to region, and this may (in part) explain the diversity of Kv expression and activity in astrocytes.

Potassium-dependent activity is an integral part of CNS function, and astrocytes play a key role in regulating its levels. Throughout the CNS, astrocytes express these K^+^ channels to varying degrees, demonstrating the physiological heterogeneity and diversity of these cells. The functional outcomes of these channel profile differences have not been completely elucidated, but several studies have found a correlation between the expression of particular K^+^ channels and cell proliferation (Bordey et al., 2001). Additionally, given that high extracellular K^+^ has been linked to neuronal damage, the heterogeneity of astrocytic K^+^ channels may represent regional and subregional susceptibility to K^+^-induced neuronal damage. This susceptibility may be particularly exacerbated in times of stress or injury; it is possible that these functional differences may only be observed under reactive conditions, or when the system has been perturbed significantly.

## Na^+^ Channels Are Not Only Expressed in Neurons

A classic dogma is that voltage-dependent sodium (Na^+^) channels are only associated with excitable cells, such as neurons, because of their role in driving an action potential. Upon membrane depolarization, these voltage-dependent Na^+^ channels open and allow a transient inward Na^+^ current, which marks the initiation of the action potential (Pappalardo et al., 2016). However, these voltage-dependent Na^+^ channels have also been identified on non-excitable cells, such as Schwann cells, microglia, and astrocytes (Black and Waxman, 2013; Pappalardo et al., 2016). Though these voltage-dependent Na^+^ channels are not responsible for action potential initiation in astrocytes, they are thought to play an important role in some astrocytic functions, particularly ion and neurotransmitter homeostasis and reactive astrogliosis (Parpura and Verkhratsky, 2012; Pappalardo et al., 2016; Verkhratsky et al., 2019). Intracellular sodium transients in hippocampal astrocytes have been observed following Schaffer collateral stimulation, suggesting sodium signaling in astrocytes occurs in response to excitatory synaptic activity (Langer and Rose, 2009). Thus, understanding the heterogenous nature of astrocytic Na^+^ signaling and channel expression may provide critical insight into astrocyte function.

Voltage-dependent Na^+^ channels consist of an α-subunit, of which there are nine isoforms (Na_v_1.1–Na_v_1.9) and a β-subunit of which there are only four isoforms (Pappalardo et al., 2016; Verkhratsky and Nedergaard, 2018). Astrocyte expression of these voltage-gated Na^+^ channels has been noted in regions such as the spinal cord, cerebellum, optic nerve, cortex, and hippocampus (Sontheimer et al., 1991; Black et al., 1995; Kressin et al., 1995; Reese and Caldwell, 1999; Schaller and Caldwell, 2003; Ziemens et al., 2019; Rurak et al., 2020). Sodium signals have also been measured in astrocytes of the white matter (Moshrefi-Ravasdjani et al., 2017). In this study, Na^+^ transients, as determined by changes in the fluorescence of the sodium indicator, SBFI, were observed following glutamate application (Moshrefi-Ravasdjani et al., 2017).

The (gene) expression of these Na_v_ subtypes has been noted to change across development, at least in cortical astrocytes (Rurak et al., 2020). In this study, the authors combined translating ribosome affinity purification with RNA sequencing (TRAPseq) to measure gene expression changes specifically in cortical astrocytes. Across development, there was significant differential expression of several genes that encode for voltage-gated Na^+^ channels, including SCN1A (which encodes for the Na_v_1.1 subtype), SCN3A (Na_v_1.3), SCN8A (Na_v_1.6), and SCN11A (Na_v_1.9; Rurak et al., 2020). Though these differentially expressed genes were not validated using alternative methods (i.e., RT-qPCR), they do suggest that the physiological properties of astrocytes (such as Na^+^ channels and signaling) are highly dynamic across development, and extrapolating data to other studies must be done with caution.

### Na^+^ Channel Subtype Expression Is Heterogenous in Astrocytes

Several of these Na^+^ channel subtypes have been observed in this glial population, including Na_v_1.2, Na_v_1.3, Na_v_1.5, and Na_v_1.6 (Black et al., 1995; Schaller and Caldwell, 2003; Black and Waxman, 2013; Pappalardo et al., 2016). However, there is considerable heterogeneity in Na_v_ subtype expression in astrocytes across the CNS. For example, one study evaluated the expression of Na_v_ 1.2 and Na_v_ 1.3 in cultured astrocytes of the spinal cord and optic nerve (Black et al., 1995). Immunocytochemistry revealed higher levels of Na_v_ 1.2 and Na_v_ 1.3 in spinal cord astrocytes compared to those of the optic nerve (Black et al., 1995). Furthermore, the researchers noted varying expression of these Na^+^ channels in morphological subtypes of astrocytes. In particular, spinal cord astrocytes classified as “stellate” had moderate levels of Na_v_ 1.2 but the “flat” astrocytes of this region expressed only low levels of the channel. Similarly, in the optic nerve, the “stellate” astrocytes expressed low levels of Na_v_ 1.2, but this expression was negligible in astrocytes classified as “flat” (Black et al., 1995). This study demonstrates the heterogeneity of some voltage-dependent Na^+^ channel subtypes across the CNS and further demonstrates how there is heterogenous expression within astrocytes of a given region. Data from this study suggests astrocyte morphology may be correlated to the diversity of Na_v_ subtype expression.

Another study in the cerebellum found heterogeneity in Na_v_ expression amongst morphological subtypes of astrocytes in this region. In the cerebellum, Na_v_1.6 expression has been noted in the processes of Bergmann glia, but the subtype was not identified in astrocytes of the granule cell layer (Schaller and Caldwell, 2003), suggesting again that morphological subtypes of astrocytes express different voltage-dependent Na^+^ channels. Interestingly, high expression of Na_v_1.6 was observed in cerebellar granule cells themselves (Schaller and Caldwell, 2003), implying that Na_v_ expression in astrocytes may, in part, be driven by the expression of these channels in neighboring neurons. Like with the K^+^ channels discussed above, it is probable that the heterogenous expression of voltage-dependent Na^+^ channels in astrocytes is the result of the interplay between various intrinsic and extrinsic factors. Understanding all these different influences will help uncover the molecular and electrophysiological underpinnings of the functional differences across astrocytes.

The heterogenous expression of Na_v_1.6 may have important implications about an astrocyte’s ability to respond appropriately to injury. Zhu et al. (2016) demonstrated that Na_v_1.6 expression is significantly upregulated in hippocampal astrocytes following status epilepticus in the kainic acid model of epilepsy and that this upregulation is strongly correlated to the severity of both the seizures and the reactive astrogliosis. Interestingly, the authors noted several lines of evidence demonstrating large voltage-dependent Na^+^ currents in reactive astrocytes following seizure (de Lanerolle and Lee, 2005), suggesting that the increase in Na_v_1.6 in reactive astrocytes may contribute to hyperexcitability in an epileptic brain (Zhu et al., 2016). This may be caused by a Ca^2+^-mediated release of glutamate from astrocytes. Upregulation of Na_v_1.6 could drive increases in Na^+^ influx, which, *via* the Na^+^/Ca^2+^ exchanger, could subsequently induce increases in intracellular Ca^2+^ levels, and Ca^2+^-mediated activities like the release of glutamate (Zhu et al., 2016). In an epiletic brain, this mechanism might further drive hyperexcitability and epileptogenesis. It is therefore possible that baseline differences in Na_v_1.6 expression in astrocytes might be indicative of the potential risk for hyperexcitability following seizure activity.

Like Na_v_1.6, the Na_v_1.5 subtype may play an important role in reactive astrogliosis. Knockdown of Na_v_1.5 mRNA in primary rat cortical astrocytes resulted in impaired wound closure following a scratch injury (Pappalardo et al., 2014). Furthermore, the scratch injury induced an intracellular Ca^2+^ response that was attenuated by the Na_v1.5_ knockdown (Pappalardo et al., 2014).

Changes in astrocytic Na_v_1.5 expression have also been observed in other models of CNS injury. In one study, conditional knockouts lacking Na_v1.5_ in astrocytes were generated. Compared to wildtype animals, the conditional knockouts developed more severe clinical outcomes in an EAE model of Multiple Sclerosis, though this effect was only observed in female mice (Pappalardo et al., 2018). This suggests that Na_v_1.5 may be important in mediating the astrocytic response to pathological conditions, though these effects may be sex-specific. Interestingly, some of the astrocytes lacking Na_v_1.5 appeared to have a more simple morphology compared to those of wildtype animals (Pappalardo et al., 2018).

### Astrocytic Na^+^ Currents Are Linked to Specific Morphologies

The differences in Na_v_ subtype expression likely underlies variation in astrocytic Na^+^ currents and associated pharmacology. Several studies have demonstrated heterogeneity of Na^+^ currents in two morphological subclasses of astrocytes (Sontheimer and Waxman, 1992; Sontheimer et al., 1994), suggesting different (morphological) subclasses of astrocytes may express different densities of the various Na_v_ subtypes. Fibrous astrocytes exhibit Na^+^ currents comparable to neurons; they tend to activate at relatively depolarized potentials and inactivate rapidly. Protoplasmic astrocytes, on the other hand, tend to activate at relatively negative potentials and inactivate more slowly. Additionally, different morphological classes of astrocytes have distinct sensitivities to tetrodotoxin, a blocker of a subset of sodium channel isoforms including Na_v_1.1–1.4, Na_v_1.6, and Na_v_1.7 (Sontheimer et al., 1994). In the spinal cord, the Na^+^ currents of astrocytes characterized as “stellate” (with numerous processes) were highly sensitive to tetrodotoxin but those that had a flat or “pancake” morphology exhibited Na^+^ currents that were largely resistant to the drug (Sontheimer and Waxman, 1992), highlighting the extensive diversity of the astrocyte population. This suggests morphological subtypes of astrocytes may express different Na^+^ channel subtypes, further emphasizing the heterogeneity of this cell population.

Heterogeneous astrocytic Na^+^ signals have also been observed in those from different regions, particularly the neocortex and hippocampus (Ziemens et al., 2019). Neocortical astrocytes exhibited larger intracellular Na^+^ transients following glutamate application in slice compared to those of the hippocampus (Ziemens et al., 2019).

The full extent to which the expression of these sodium channels and currents vary between astrocytes throughout the CNS has not been fully delineated but is imperative given the current evidence which suggests voltage-dependent Na^+^ channels are involved in several critical astrocytic functions such as the regulation of ion channels and in response to CNS injury such as epileptogenesis (Qiao et al., 2013; Zhu et al., 2016). It is already established that Na^+^ currents appear to differ between morphological subtypes of astrocytes but whether these properties also differ between other subtypes of astrocytes remains to be explored. Nonetheless, the differences in Na_v_ subtype expression and the biophysical and pharmacological properties of Na^+^ currents further demonstrate the electrophysiological heterogeneity of astrocytes.

## Multiple Ca^2+^ Signaling Pathways in Astrocytes, An Integral Part of Astrocyte Physiology

Traditionally, astrocytes were believed to be passive cells whose sole function was to provide support for neuronal function. However, seminal studies in the 1990s revealed that astrocytes are capable of responding to synaptic activity through increases in intracellular calcium [Ca^2+^]_i_ levels (Porter and McCarthy, 1995; Pasti et al., 1997; Kang et al., 1998; Agulhon et al., 2008). These effects are also seen in cultured human astrocytes (Navarrete et al., 2013; Hashioka et al., 2014). Since then, calcium signaling cascades have been recognized as an integral part of astrocyte physiology. Importantly, under physiological conditions, both spontaneous and receptor-activated Ca^2+^ signals have been observed in astrocytes (Shigetomi et al., 2019).

Astrocytes express a vast array of G-protein coupled receptors (GPCRs; Shigetomi et al., 2019). In particular, Gα_q_-linked GPCRs (G_q_PCRs) are coupled to internal Ca^2+^ stores and may be the specific link between changes in [Ca^2+^]_i_ in response to neurotransmitter release at the synapse (Agulhon et al., 2008). Activation of a G_q_PCRs leads to the hydrolysis of the membrane lipid phosphatidylinositol 4,5-biphosphate (PIP_2_) *via* the enzyme phospholipase C (PLC; Agulhon et al., 2008). This produces diacylglycerol (DAG) and inositol 1,4,5-trisphosphate (IP_3_), the latter which binds to and activates IP_3_ receptors on the endoplasmic reticulum membrane, thereby releasing Ca^2+^ from intracellular stores (Agulhon et al., 2008).

There are three isoforms of the IP_3_ receptor (IP_3_R; Sherwood et al., 2017). In astrocytes, the IP_3_R_2_ subtype appears to be the primary receptor subtype driving the PLC/IP_3_ pathway (Holtzclaw et al., 2002; Sheppard et al., 2002; Petravicz et al., 2008); deletion of IP_3_R_2_ resulted in a lack of spontaneous and G_q_PCR-induced elevations in Ca^2+^ in astrocytes (Petravicz et al., 2008). However, some studies have demonstrated Ca^2+^ signals still occur in the astrocytes of mice lacking the IP_3_R_2_ subtype (Stobart et al., 2018b), suggesting other mechanisms contribute to intracellular Ca^2+^ astrocytic signaling. In fact, a recent study found that the first and third isoforms of IP_3_R may also contribute to Ca^2+^ signaling in astrocytes, albeit to a lesser extent (Sherwood et al., 2017).

Nonetheless, the IP_3_R_2_ subtype plays a particularly important role in Ca^2+^ signaling. A study from Holtzclaw et al. (2002) demonstrated distinct regional astrocytic subcellular expression of IP_3_R_2_. Astrocytes of the hippocampus expressed IP_3_R_2_ in their somata and processes, though there appeared to be a higher density in the large and fine processes. This was also true for cerebellar Bergmann glia in the molecular layer, which had punctate expression of IP_3_R_2_ in their finer processes. In contrast, Bergmann glia of the Purkinje cell layer expressed IP_3_R_2_ primarily in their somata (Holtzclaw et al., 2002). The study also showed that IP_3_R_2_ is particularly enriched in astrocytic processes that encircle synapses, highlighting an important putative role of Ca^2+^ signaling in mediating the link between neurotransmission and astroglia.

### Spontaneous Ca^2+^ Oscillations in Astrocytes

Spontaneous Ca^2+^ signaling events occur in the absence of neuronal activity (Parri et al., 2001) and have been observed in astrocytes throughout the CNS including in the hippocampus (Nett et al., 2002; Rungta et al., 2016), cortex, striatum, and thalamus (Parri et al., 2001; Aguado et al., 2002; Jiang et al., 2014); the presence of spontaneous Ca^2+^ signals have also been confirmed, at least *in vitro*, in human astrocytes (Navarrete et al., 2013). They have been observed in Bergmann glia of the cerebellum (Aguado et al., 2002) as well as in astrocytes of the olfactory bulb (Otsu et al., 2015). Both extracellular and intracellular Ca^2+^ levels are critical for driving these spontaneous oscillations (Aguado et al., 2002); removal of extracellular Ca^2+^ is sufficient to prevent spontaneous Ca^2+^ signals *in vitro* (Aguado et al., 2002) and the loss of the IP_3_R_2_ reduces spontaneous events *in vivo* (Petravicz et al., 2008; Jiang et al., 2016; Yu et al., 2018). However, some reports have noted spontaneous Ca^2+^ events even in the astrocytes of IP_3_R_2_^−/−^ mice (Sherwood et al., 2017), suggesting that these mechanisms are not the only ones that drive spontaneous Ca^2+^ signals in astrocytes. Recent evidence from Wu et al. (2019) suggests that the morphology of astrocytes may be an important factor in driving these spontaneous Ca^2+^ signals- these events were more frequent in thin processes with a high surface-to-volume ratio. Given the extensive morphological heterogeneity of astrocytes, it is possible that the localization of transient Ca^2+^ events to thinner processes can explain some of the diversity in astroglial calcium signaling, particularly spontaneous events. The spontaneity of these Ca^2+^ signals fluctuate between astrocytes across the CNS, perhaps reflecting diversity in some of these processes. For example, hippocampal astrocytes, specifically of the CA1 *stratum radiatum* region, have a higher frequency of spontaneous Ca^2+^ events than those of the dorsolateral striatum (Chai et al., 2017), but fewer oscillations than those of the cortex (Navarrete et al., 2013), perhaps reflecting regional differences in the mechanisms that drive these spontaneous events.

In the hippocampus, a subset of astrocytes displayed no spontaneous Ca^2+^ signals, but of the astrocytes that did, there was significant variability in the frequency of these events (Nett et al., 2002). Some had fairly frequent Ca^2+^ oscillations at intervals between 0.5 and 2 min, whilst others had much more irregular intervals between oscillations, with intervals that exceeded 2 mins (Nett et al., 2002). Similar heterogeneity has been observed in the thalamus; the majority of thalamic astrocytes showed multiple spontaneous Ca^2+^ events over a 10-min period but several displayed only one spontaneous Ca^2+^ signal in the same time (Parri et al., 2001). In the somatosensory cortex, astrocytes from layer I had nearly double the frequency of spontaneous Ca^2+^ activity compared to those from layer II/III (Takata and Hirase, 2008).

Precisely what the differences in spontaneous Ca^2+^ signaling across astrocyte subpopulations mean has yet to be fully elucidated. One study found that hippocampal astrocytes which did not exhibit spontaneous Ca^2+^ signals did not differ in morphology or electrophysiology from those that did (Nett et al., 2002), demonstrating that the classification of astrocytes is not so straightforward as one morphological class displaying one set of electrophysiological properties, or molecular markers. Regardless, it is plausible that the differences in these spontaneous Ca^2+^ oscillations represent important distinctions between the various types of astrocytes. Astrocytes displaying no spontaneous Ca^2+^ are still able to respond to some neurotransmitter release (Nett et al., 2002), but the lack of spontaneous Ca^2+^ signals might indicate a decreased ability or less sensitive response to these types of events which normally evoke a Ca^2+^ response.

The mobilization of both intracellular and extracellular Ca^2+^ may be another important mechanism driving spontaneous Ca^2+^ events (Aguado et al., 2002). Removal of extracellular Ca^2+^ blocked spontaneous Ca^2+^ events in hippocampal astrocytes in slices (Aguado et al., 2002). Likewise, when thapsigargin, a drug which inhibits endoplasmic reticulum Ca^2+^-ATPase activity and thus depletes ER Ca^2+^ stores, was applied to hippocampal slices, spontaneous Ca^2+^ activity in astrocytes was blocked. This suggests that intracellular Ca^2+^ stores are also necessary for spontaneous Ca^2+^ events in astrocytes (Aguado et al., 2002). The lack of spontaneous Ca^2+^ activity in astrocytes might therefore reflect a depletion of extracellular or intracellular Ca^2+^ signaling capacity, highlighting the influence of both extrinsic and intrinsic factors in the development of astroglial spontaneous Ca^2+^ activity. As with the frequency, the amplitude of these spontaneous astrocyte Ca^2+^ transients are variable- both inter-and intra-regionally (Aguado et al., 2002). This is true in several regions including the cortex, hippocampus, thalamus, hypothalamus, and spinal cord (Aguado et al., 2002). The heterogeneity of these spontaneous events is likely the result of regional and subregional diversity in the presence of extracellular Ca^2+^ and the available intracellular Ca^2+^ stores in astrocytes.

It is clear that there are subtle differences between astrocytes displaying spontaneous Ca^2+^ oscillations and those that do not (Nett et al., 2002). Furthermore, it is clear that of the astrocytes that do exhibit spontaneous Ca^2+^ oscillations, they differ significantly in several of their properties including the frequency and the amplitude of these signals. It is possible these distinct spontaneous Ca^2+^ oscillations have extensive functional consequences, making the understanding of this heterogeneity pertinent to comprehending the role of astrocytes within the CNS.

### Astrocytic Ca^2+^ Signals Differ in Subcellular Compartments

Initial studies into astrocytic Ca^2+^ signaling focused on cytosolic Ca^2+^ signals, primarily in the somata of astrocytes, but the use of genetic encoded calcium indicators (GECIs) have enabled higher resolution visualization of localized Ca^2+^ transients in the processes and finer processes of astrocytes (Shigetomi et al., 2013; Stobart et al., 2018a; for a comparison of calcium imaging techniques, see Smith et al., 2018). For example, spontaneous Ca^2+^ transients have been observed in the somata, processes, and fine processes of striatal astrocytes (Jiang et al., 2014). These cytosolic and local transients have also been observed in astrocytes of the cortex (Agarwal et al., 2017), hippocampus (Zur Nieden and Deitmer, 2006; Jiang et al., 2014), somatosensory cortex (Wang et al., 2006), and olfactory bulb (Otsu et al., 2015).

Like the extensive inter-and intraregional variability in the frequency and amplitude of astrocytic Ca^2+^ signals, there is substantial data suggesting that these events are also variable across the distinct subcellular compartments of astrocytes. In particular, it seems that spontaneous Ca^2+^ events occur more frequently in the processes compared to the somata of astrocytes. One study found the majority of Ca^2+^ events (~85%) were localized to the processes, with a much smaller percentage (~10%) in the endfeet, and an even smaller percentage (~5%) in the somata (Bindocci et al., 2017). A very similar distribution of spontaneous Ca^2+^ events was seen in astrocytes within the somatosensory cortex, with approximately 80% of those signals occurring in the fine processes (Kanemaru et al., 2014). In astrocytes of the *stratum lucidum* region of the hippocampus, spontaneous Ca^2+^ signals were virtually absent in the somata but were frequently observed in the processes (in fact, there was about an 8-fold greater increase in these events in the processes compared to the somata; Haustein et al., 2014). The high frequency of spontaneous Ca^2+^ transients in the fine processes of astrocytes relative to their somata has also been observed in the visual cortex (Asada et al., 2015).

The spontaneous Ca^2+^ transients observed throughout the subcellular compartments of astrocytes also differ in their ability to spread from the source event. In the CA1 region, for example, researchers identified distinct Ca^2+^ fluctuations; one fluctuation produced a wave-like property, spreading to adjacent areas, whereas the other produced a restricted response which the authors referred to as a microdomain (Srinivasan et al., 2015). Both types of fluctuations were displayed in the processes, and each differed from fluctuations measured from the somata (Srinivasan et al., 2015).

Spontaneous Ca^2^ transients, while common in astrocytes, vary tremendously in the frequency, amplitude, and even type of fluctuation they produce. These properties differ at the regional, subregional, and subcellular levels. The current literature highlights the complexity of astroglial Ca^2+^ signaling at the regional, sub-regional, and cellular level.

This subcellular heterogeneity of Ca^2+^ signals, combined with the varied expression of voltage-dependent and voltage-independent ion channels (such as those discussed previously), suggests that astrocytes possess different electrophysiological profiles amongst distinct microdomains of the cell. Thus, permitting a localized response to environmental changes. These differences in subcellular compartments may also explain voltage-dependent ion channels in astrocytes; while the relatively low membrane resistance and hyperpolarized phenotype would (generally) require highly depolarizing events to activate these channels, the intracellular heterogeneity means there could be local variability in the membrane resistance and RMP of the processes, fine processes, and soma of the astrocyte.

## Astrocyte Physiology Influences Synaptic Networks

Thus far, we have summarized the available data on the heterogeneity of astrocyte physiology. But what do these differences mean for the greater network? Does this heterogeneity translate to functional differences between other cells in the network such as neurons? If, and how, these differences may influence the network is not fully understood, but there is some evidence to suggest they can.

K^+^ homeostasis is a critical function of astroglia, but they are not the sole cell type that can buffer this ion. Neurons, too, are able to buffer against K^+^. Not all astrocytes may regulate K^+^ equally; for example, the expression of the K_ir_4.1, a key channel in buffering K^+^, is highly variable amongst astrocytes. In areas where the K^+^ buffering capacity of astrocytes is low, then neurons may need to be more active at regulating K^+^ to help offset that deficit. This increased activity of neuronal transporters could drive higher energy and metabolic demands, which might, in turn, make these neurons more susceptible to electrical and chemical perturbations. The implications of this heterogeneity in K^+^ buffering are not yet understood, but moving forward may be particularly important when considering why some neuronal populations are more susceptible to perturbations and damage than others.

Several studies have shown a “wave-like” phenomena between astrocytes; that is, a Ca^2+^ signal appears in one astrocyte followed quickly by a Ca^2+^ signal in another nearby (Verkhratsky, 2006). This is mediated through the extensive gap junction coupling of astrocytes, forming the “net-like” structure of the glial syncytium and demonstrates how astrocytes can influence surrounding astrocytes. Of course, Ca^2+^ signals are only one way astrocytes can influence each other; ions (such as K^+^—see “Astroglial K^+^ Spatial Buffering Mediated Through K_ir_4.1 Subtype” section) can also cross gap junctions as can energy substrates like glucose (Rouach et al., 2008) and lactate (Murphy-Royal et al., 2020).

It has also been theorized that astrocytic Ca^2+^ signals can influence synaptic activity. One study found that spontaneous Ca^2+^ signals in hippocampal astrocytes were localized to specific regions in their processes (Nett et al., 2002). These “microdomains” were typically asynchronous, leading the authors to believe the astrocytes might be influencing neurons in a synapse-specific manner. However, they did note some “wave-like” activity between processes of the same astrocyte, suggesting a potential larger influence on the greater synaptic field (Nett et al., 2002). A recent study did find a more direct effect of the glial syncytium on synaptic activity (Murphy-Royal et al., 2020). In this experiment, an acute stressor was sufficient to reduce gap junction coupling between astrocytes, leading to a decrease in energy substrate transport between astrocytes and subsequent impairment in LTP (Murphy-Royal et al., 2020), clearly demonstrating that an intact glia syncytium is necessary for synaptic plasticity.

## Optogenetics and DREADDs: Physiologically Relevant for The Study of Astrocytes?

Understanding the physiology of astrocytes has been challenging partly because of a lack of technology sensitive enough to measure many of these properties. This has been compounded by the inability to directly target and manipulate select populations of astrocytes *in vivo*. In the past couple of decades, the advent of novel technologies has enabled specific cell populations, such as astrocytes, to be selectively targeted and manipulated, thus permitting more in-depth analysis of their function within the CNS. In particular, optogenetics and DREADDs have been frontrunners for targeting astrocytes.

With optogenetics, cells of interest are targeted with light-sensitive proteins, known as opsins, which generally exist as ion channels and pumps (Bang et al., 2016). The absorption of a specific wavelength by the opsin induces a conformational change, driving electrical changes across the plasma membrane (Bang et al., 2016). These cellular changes differ extensively depending on the type and variant of opsin used. Channelrhodopsin 2 (ChR2), for example, is a cation channel that is activated specifically by 473 nm (blue) light (Nagel et al., 2003), leading to an influx of cations (namely protons and Na^+^) that produces membrane depolarization (Boyden et al., 2005). In astrocytes, the cation influx (particularly of Na^+^) may be important for modulating neuron homeostasis and GABAergic transmission (Parpura and Verkhratsky, 2012). Na^+^ signaling in astrocytes (particularly Na^+^ influx mediated by voltage-dependent Na^+^ channels) is thought to be necessary for the activity of the Na^+^/K^+^ ATPase pump (Verkhratsky et al., 2019), suggesting a role in the maintenance of ion homeostasis. Additionally, intracellular Na^+^ is important in controlling GABA uptake *via* the GABA transporter (GAT) pathways; decreases in Na^+^ can reverse GAT-dependent transport causing GABA to be released (Verkhratsky et al., 2019). However, ChR2 can also allow the influx of Ca^2+^, which has been shown to cause the release of gliostransmitters like ATP (Gourine et al., 2010; Chen et al., 2013) and glutamate (Haydon and Carmignoto, 2006). ChR2 stimulation in astrocytes has been reliably shown to induce changes in intracellular Ca^2+^ (Li D. et al., 2012; Figueiredo et al., 2014; Perea et al., 2014; Mederos et al., 2019; Balachandar et al., 2020); these changes have been observed to enhance both excitatory and inhibitory synaptic transmission in the primary visual cortex (Perea et al., 2014). The use of ChR2 to activate astrocytes has also been shown to induce the release of ATP (Gourine et al., 2010; Figueiredo et al., 2014).

Halorhodopsin, on the other hand, is optimally activated by approximately 590 nm (yellow) light and drives hyperpolarization of cells through the influx of Cl^−^ ions (Bang et al., 2016). Cl^−^ has been shown to affect outward K^+^ currents in cultured astrocytes (Bekar and Walz, 1999), showing that the ion has an important role in mediating astrocyte physiology. Intracellular Cl^−^ in astrocytes has also been theorized to play a role in mediating crosstalk between neurons and astrocytes because of its impact on several astrocytic transporters, including the excitatory amino acid transporters EAAT1 and EAAT 2 (GLAST and GLT-1 in rodents, respectively), GAT 1 and 3, and NKCC1 (Wilson and Mongin, 2019). Changes in astrocyte intracellular Cl^−^ levels are associated with EAAT activity and the movement of Cl^−^ across the membrane is necessary for GAT function (Wilson and Mongin, 2019). Given the apparent role of Cl^−^ signaling in astrocyte physiology, the manipulation of this ion may be important for deepening our understanding of Cl^−^ function within an astrocyte. Moving forward, the use of halorhodopsin in astrocytes will likely become an invaluable tool. As with neurons, the functional consequences of the use of different opsins will vary significantly in astrocytes.

Like halorhodopsin, archaerhodopsin (Arch) and archaerhodopsin-T (Arch-T) also respond to yellow or green light (around 532 nm) and drive hyperpolarization of a cell (El-Gaby et al., 2016). However, unlike halorhodopsin, Arch and Arch-T induce hyperpolarization through the efflux of protons (El-Gaby et al., 2016; Poskanzer and Yuste, 2016). This hyperpolarization is sufficient to induce changes in intracellular Ca^2+^ in astrocytes; work from Poskanzer and Yuste (2016) showed that Arch activation in cortical astrocytes evoked Ca^2+^ transients. These transients appeared primarily in the branches and lasted approximately the same length as those generated from spontaneous events in non-stimulated controls (Poskanzer and Yuste, 2016). However, a slight decrease in the amplitude of these Ca^2+^transients was noted in the Arch-stimulated compared to the non-stimulated astrocytes (Poskanzer and Yuste, 2016), suggesting that optogenetic stimulation may recapitulate some, but not all, physiological properties of astrocytes. Which of these physiological properties are conserved may depend on the type of opsin used, the region of interest, and the original physiological state of the astrocyte.

Melanopsin responds to shorter wavelengths of light and is optimally activated by light of about 420–450 nm (blue) light (Newman et al., 2003; Wang et al., 2019). It is slightly different from other opsins as it binds to a GPCR, leading to Ca^2+^ signaling *via* activation of the PLC/IP_3_ pathway (Mederos et al., 2019). As such, it has been suggested as a good tool for investigating the physiological effects of astrocytes. One recent study transfected melanopsin into hippocampal astrocytes and found that stimulation resulted in robust IP_3_-dependent Ca^2+^ signals in their fine processes and the release of ATP/adenosine at the synapse (Mederos et al., 2019).

Adaptations to these opsins (particularly ChR2) have been generated in recent years, including ChETA and step function opsins (SFO; Bang et al., 2016). In neurons, ChETA has been shown to induce ultrafast spiking and SFOs are known to produce prolonged, sub-threshold membrane depolarizations (Bang et al., 2016) but how these differences might translate to astrocyte activity is not well characterized.

Optogenetics provides precise spatial and temporal resolution for the manipulation of cells within the CNS. The high spatial resolution can be obtained *via* the viral (i.e., adeno-associated virus or lentivirus) transfection of the genetically engineered opsin to a targeted region of interest. Combined with a cell-type-specific promoter, a high degree of cell specificity is attainable. The ability to precisely modulate the frequency of light pulses delivered to these regions of interest allows extensive manipulation of these cells, and thus provides a high degree of temporal resolution that is not seen in many other technologies. Optogenetics has been particularly valuable for studying neuronal function throughout the CNS, and in more recent years, the technology is being applied to astrocytes. Since the effects of depolarization and hyperpolarization on astrocyte signaling are not as well understood as these effects in neurons, the relevance and generalizability of optogenetic approaches to manipulate and understand astrocytes remains to be fully elucidated.

The use of designer receptors exclusively activated by designer drugs (DREADDs) provides an alternative to target cells, particularly astrocytes. DREADDs represent one of the most commonly employed forms of chemogenetics, in which genetically engineered receptors selectively bind ligands to drive transient changes (usually activation or inactivation) in the region of interest (Roth, 2016; Smith et al., 2016). The genetically engineered GPCRs have little to no response to endogenous ligands but a strong response to synthetic ones that are otherwise biologically inert (Bang et al., 2016). When the ligand (or actuator) is introduced, it binds to and activates the receptor, activating the downstream Gq, Gi, or Gs pathways that are coupled with these receptors.

Activation of neuronal and non-neuronal cells is mediated through DREADDs targeting the Gq signaling pathway, of which the hM3Dq is most frequently used (Alexander et al., 2009; Roth, 2016). Inhibitory DREADDs, which silence neuronal activity, target the Gi pathway, the most common being the hM4Di receptor (Roth, 2016). Traditionally, clozapine-N-oxide (CNO) has been used as the chemical actuator for both hM3Dq and hM4Di (as well as several other DREADDs), but new evidence suggesting it can reverse-metabolize to its parent compound, clozapine (Manvich et al., 2018; Walker and Kullmann, 2020), and that it may have poor penetrance into the brain (Bonaventura et al., 2019) has led to the use of newer classes of chemical actuators including Compound 13, Compound 21, JHU37152, and JHU37160 (Bonaventura et al., 2019).

Like opsins, these receptors can be placed under the control of cell-specific promoters, restricting expression to a particular group of cells. The use of a fluorescent tag such as green fluorescent protein (GFP) allows for easy visualization of the cells expressing the opsin or DREADD of interest.

Since DREADDs use intracellular Ca^2+^ signaling to modulate cell activity, many have suggested they are particularly useful for studying astroglial activity given the integral role of Ca^2+^ signaling in astroglial function and communication; that is, the manipulation of astrocytes *via* the GPCR activation might be physiologically relevant. However, neither DREADDs nor optogenetics may be physiologically relevant for the study of astrocytes unless the unique physiological features of astrocytic subtypes are addressed in the experimental design.

Due to their low membrane resistance, astrocytes will likely elicit smaller current changes to optogenetic stimulation than neurons. As such, “stronger” or more “intense” stimulation may be needed to elicit a response in astrocytes. Fine-tuning of experimental guidelines is needed to determine what parameters would elicit a physiological response in astrocytes.

**Figure 2:**
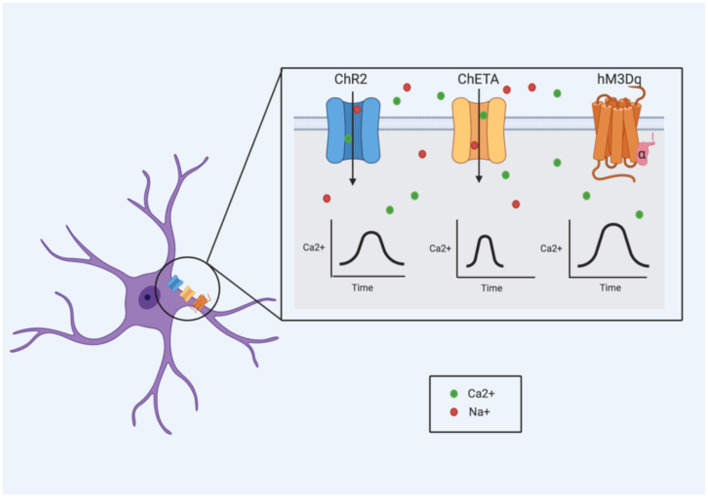
Ca^2+^ signaling dynamics are influenced by the mechanism of Ca^2+^ induction. Therefore, opsin and designer receptors exclusively activated by designer drug (DREADD) variants that utilize alternative mechanisms to exert their effects may also show varying Ca^2+^ responses. These ion channel or receptor kinetics need to be considered when determining the best approach for targeting an astrocyte population.

Another important consideration for the manipulation of [Ca^2+^]_i_ in astrocytes is that the mechanism which induces a rise in [Ca^2+^]_i_ can influence the speed of the change in these levels (Bazargani and Attwell, 2016). This suggests that the use of opsin variants (such as channelrhodopsin 2, ChR2, and its variant ChETA), which have similar, but differing mechanisms (ChETA has faster kinetics and in neurons has been proven to have more rapid repolarization than ChR2; Gunaydin et al., 2010), may induce changes in [Ca^2+^]_i_ at varying speeds, possibly contributing to alternative downstream effects. This is further compounded by the regional and subregional diversity of astrocyte physiology previously discussed in this review, rendering rigorous pilot studies essential prior to the use of DREADDs or optogenetics to target astrocytes (see Figure 2).

One study compared several ChR2 variants; all four variants evaluated were sufficient to induce large increases in [Ca^2+^]_i_ (Figueiredo et al., 2014). There were no significant differences in the overall [Ca^2+^]_i_ responses or in the dynamics of these responses between the ChR2 variants (Figueiredo et al., 2014). However, another study found the Ca^2+^-translocating channelrhodopsin (CatCh) variant was more efficient than ChR2 in controlling Ca^2+^ elevations in astrocytes (Li D. et al., 2012). This implies that generalizing results from studies that employ different opsin variants may be difficult. Selecting the appropriate opsin variant to employ (i.e., to produce a particular/desired effect) will be further complicated by the diverse nature of astrocytic Ca^2+^ signaling.

Differences between opsin types rather than just opsin variants may also induce significantly different effects. A recent study compared the effects of stimulating hippocampal astrocytes with ChR2 and melanopsin (Mederos et al., 2019). Although melanopsin was more efficient at inducing Ca^2+^ signals in both the soma and fine processes (referred to as microdomains in this study) of astrocytes, ChR2 stimulation elicited greater amplitude in miniature EPSCs of CA1 pyramidal neurons (Mederos et al., 2019). This effect on synaptic activity also persisted for longer following the ChR2 stimulation. However, these changes were only seen under certain stimulation parameters and not others (Mederos et al., 2019). Despite being “depolarizing” opsins, both melanopsin and ChR2 elicit different Ca^2+^ signals and ultimately, different downstream effects. This makes direct comparisons between studies that utilize different opsins difficult and also highlights how opsins may be physiologically relevant for some contexts, but not others. The effects that different opsins have on Ca^2+^ dynamics may be important for experimental design. Consideration of the effects that different opsins have on intracellular K^+^ and Na^+^ levels is also critical given their roles in numerous astrocyte functions.

In studies where the activity of select cell populations are directly targeted and modulated, it is essential to validate that the expression of the opsin or DREADD is restricted to that population only. Co-localization of the fluorescent tag with a cell-specific marker can confirm the specificity of this expression. Within astroglial cells, Ca^2+^ signals differ throughout their subcellular compartments, with potentially variable effects. Therefore, the precise subcellular localization of an opsin or DREADD receptor within an astrocyte may modulate the overall effect on that individual cell. Moving forward, it may be important to validate whether the subcellular expression does indeed alter functional outcomes, and what those functional outcomes might be. The physiological relevance of these technologies may therefore be better understood with the full knowledge of the cellular distribution of opsins and DREADD receptors in astrocytes.

Through gap junction proteins, astrocytes can create a network of interconnected cells. Several studies have demonstrated that calcium waves initiated in one astrocyte can propagate to others surrounding it (Verkhratsky, 2006). This phenomenon was first observed from *in vitro* culture work; these results were later confirmed in acute slices (Dani et al., 1992; Konietzko and Müller, 1994), and eventually with *in vivo* experiments (Kuga et al., 2011). Similar effects have been noted with Na^+^ signals; several studies have demonstrated the ability of astrocytes to propagate these signals from one cell to another *via* these gap junctions (Rose and Ransom, 1997; Bernardinelli et al., 2004; Langer et al., 2012; Augustin et al., 2016; Moshrefi-Ravasdjani et al., 2017). What this means for studies in which astroglial cells are manipulated remains to be seen. In most cases, it is unlikely that all astrocytes in a region of interest express the opsin or DREADD receptor. However, *via* the glial syncytium, it is possible that “activation” of one astrocyte leads to the activation of surrounding astrocytes. In one study, Ca^2+^ changes were restricted to only astrocytes expressing the opsin (Arch; Poskanzer and Yuste, 2016). However, only Ca^2+^ changes were measured, and it is possible that other ions/substrates were altered in coupled, Arch^−^ astrocytes. The potential of Ca^2+^ or other compounds to move from a stimulated astrocyte to its coupled neighbors needs to be studied under other parameters and with different opsin and DREADD variants. How far this effect could spread would likely depend on the extent of astroglial coupling within regions of interest. *In vivo* imaging of astrocytes during optogenetic stimulation or DREADD activation may help answer these questions.

Importantly, whether this stimulation of one astrocyte leads to the stimulation of other astrocytes will likely also depend on the integrity of the glial syncytium. The Murphy-Royal et al.’s (2020) study described in the previous section found acute stress was sufficient to reduce the amount of gap junction coupling between astrocytes. Therefore, the paradigm under which optogenetics is employed could significantly impact the extent to which the optogenetic stimulation of astrocytes “spreads” or influences astrocytes of the neighboring area. As such, optogenetic stimulation of astrocytes under normal, physiological conditions, when presumably the glial syncytium is intact, may have a greater impact than stimulation in a model of stress or injury where this may not be the case.

**Figure 3:**
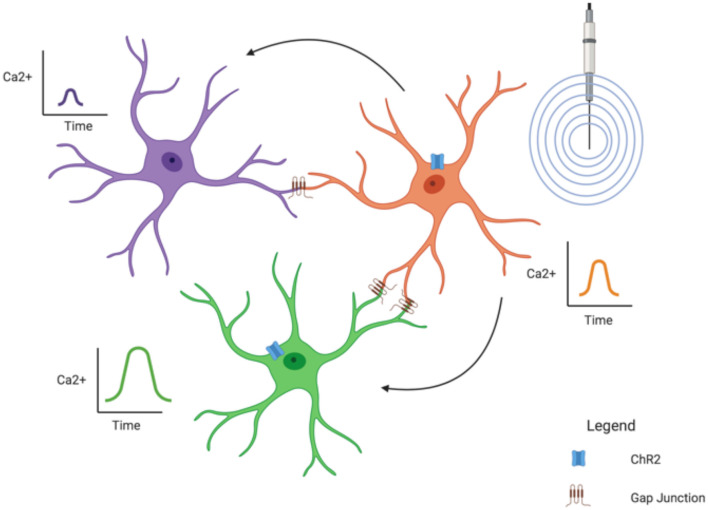
The glial syncytium may impact the physiological effects of optogenetics and DREADD technologies (optogenetics illustrated above). The connection of astrocytes *via* gap junction proteins might produce a “double stimulation” or “spillover” effect. In the diagram above, only a portion of the astrocytes (orange and green) express the opsin of interest. The green astrocyte is first stimulated following the introduction of light into the system, and then again when a neighboring astrocyte (orange) is also stimulated, generating a larger than anticipated response. In the case of “spillover,” astrocytes that do not express the opsin or DREADD in use nonetheless become “stimulated” from the activation of its neighboring astrocytes. In the image above, the purple astrocyte still exhibits a response following light stimulation despite the lack of ChR2 expression. The “stimulation” arises from the influence of the connected astrocyte (orange) that is stimulated *via* its ChR2 ion channels.

Another consideration is the potential of “double stimulation.” An astroglial cell stimulated *via* optogenetics or DREADDs could, through gap junction coupling, stimulate a neighboring astrocyte. If this astrocyte is also stimulated *via* optogenetic or DREADD technology, this may result in a “double stimulation” effect, leading to a response much greater than physiological conditions. A stronger response could mean the stimulated astrocytes influence neighboring cells, in particular, neurons, in a manner incompatible with a normal physiological response. The strength of the glial syncytium in a given area may dictate the likelihood that this happens, further highlighting the necessity of understanding the heterogeneous nature of astrocyte physiology and connectivity. As stress can influence the integrity of the glial syncytium (Murphy-Royal et al., 2020), this (potential) issue of “double stimulation” may be less of a concern in paradigms where the astrocytes are stimulated under conditions of stress or injury/disease. This demonstrates the importance of pilot studies to understand how optogenetics and DREADDs impact astoglial activity (see Figure 3).

In optogenetic and DREADD studies involving neurons, their heterogeneous nature is taken into consideration when designing experiments. The opsin variant or DREADD, for example, that is used to stimulate or inhibit a GABAergic neuron may differ from that of a glutamatergic or dopaminergic one. This same consideration needs to be taken for astrocytes. The literature shows that like neurons, astrocytes are a completely heterogenous population. They differ extensively in their morphology, electrophysiology, protein expression, and function. These subtypes are important to consider as each type may not respond in the same manner to different opsin and DREADD variants. Moving forward, understanding these subtypes will be crucial for informing experimental design. Greater knowledge of astrocyte electrophysiology will be an essential component for characterizing these astrocyte subtypes.

Similarly, in neurons, the use of an opsin or DREADD variant largely depends on the desired effect that researchers are looking to elicit. In neurons, these desired effects are typically the facilitation or inhibition of an action potential, driven by depolarization or hyperpolarization of the targeted cell. However, with astrocytes, a depolarization or hyperpolarization will not elicit these same effects. As such, understanding astrocyte physiology, and the heterogeneous nature of this physiology, will be critical for making knowledgeable and appropriate decisions regarding experimental design.

## Conclusion

The research over the past few decades has demonstrated that astrocytes are a more “excitable” and heterogenous cell population than previously believed. They differ in morphology, function, and physiology, though the extent of physiological diversity has not been fully characterized. In this review, we discussed how astrocytes exhibit a wide range of basic electrophysiological properties under basal conditions. The mechanisms underlying homeostatic potassium regulation vary between and within brain regions, as do the specific variants of K^+^ channels that mediate this K^+^ buffering. Likewise, the Na^+^ currents and Na^+^ channel subtypes that mediate those currents are also heterogenous in astrocytes. Ca^2+^ signaling, a key component of astrocyte physiology is also highly variable. There are several different types of Ca^2+^ waves present in astrocytes, and these vary considerably amongst astrocytes across the CNS. Precisely how all these electrophysiological differences influence surrounding network activity is still unclear, but there is some evidence to indicate that they do. For example, asynchronous Ca^2+^ microdomains in astrocyte processes likely provides a synapse-specific influence of network activity. This heterogeneity is important to consider for optogenetic and DREADD technologies so that the limitations and generalization of each study can be fully assessed.

Astrocytes represent a very heterogenous cell population; we have highlighted some of the vast differences between the electrophysiological properties of these cells. Despite the available evidence, there is still a substantial amount of research needed in order to truly understand the extensive diversity of this unique cell population.

## Author Contributions

JM, MH, CR, and NS wrote the manuscript. JM and NS made the figures. All authors contributed to the article and approved the submitted version.

## Conflict of Interest

The authors declare that the research was conducted in the absence of any commercial or financial relationships that could be construed as a potential conflict of interest.
